# Failure of lumbar puncture in a patient with spinal epidural lipomatosis: a case report

**DOI:** 10.1186/s40981-016-0040-y

**Published:** 2016-07-07

**Authors:** Daiki Yamanaka, Takashi Kawano, Marie Shigematsu-Locatelli, Atsushi Nishigaki, Sonoe Kitamura, Bun Aoyama, Hiroki Tateiwa, Noriko Kitaoka, Masataka Yokoyama

**Affiliations:** Department of Anesthesiology and Intensive Care Medicine, Kochi Medical School, Kohasu, Oko-cho, Nankoku, Kochi 783-8505 Japan

**Keywords:** Spinal anesthesia, Lumbar puncture, Epidural lipomatosis

## Abstract

We report a case of difficult lumbar puncture due to the inability to obtain adequate cerebrospinal fluid (CSF) in a patient later diagnosed with spinal epidural lipomatosis (SEL). A 76-year-old man with a body mass index (BMI) of 24.1 kg/m^2^ was scheduled for transurethral resection of a bladder tumor for superficial bladder cancer under spinal anesthesia. The patient had a 3-year history of inhaled steroid use for the management of chronic obstructive pulmonary disease. After placing the patient in the right lateral position, a lumbar puncture was performed *via* the median approach. However, CSF could not be tapped adequately despite repeated attempts at lumbar puncture, so general anesthetic was administered instead. Subsequently, both anesthesia and surgery proceeded uneventfully. On the first postoperative day, the patient developed mild postdural puncture headache (PDPH), which was treated conservatively. No postoperative neurological complications related to spinal anesthesia were observed. Approximately 2 months after discharge, the patient reported progressive lower back pain and was diagnosed with SEL by magnetic resonance imaging (MRI). A lumbar laminectomy and removal of excessive adipose tissue was performed. After surgery, the patient’s symptoms resolved. The pathogenesis of SEL involves excess fat tissue deposition in the spinal canal, which can lead to obliteration of the spinal subarachnoid space. Therefore, in this patient, the SEL was thought to have caused the inability to obtain adequate CSF during lumbar puncture, and was associated with difficult spinal anesthesia.

## Background

Spinal epidural lipomatosis (SEL) is characterized by pathological overgrowth of the extradural fat [1–3]. The manifestations of SEL are frequently asymptomatic or result in nonspecific back pain [1]. However, progressive spinal cord compression can lead to subsequent neurological deficits. There are no documented prevalence rates regarding SEL, but it is considered to be rare [2, 3]. SEL is most commonly associated with long-term use of exogenous steroids. However, there are additional reported risk factors, including Cushing’s disease, obesity, epidural steroid injection, hypothyroidism, pituitary prolactinoma, renal transplants, and rheumatoid arthritis. Finally, a number of idiopathic cases have also been reported [2–4].

Although many cases of SEL have been documented, only a few reports highlight the risk of neuraxial anesthetic procedures in this patient group [5, 6]. Here, we report a case of failure of lumbar puncture for spinal anesthesia most likely due to SEL that was not identified prior to surgery.

## Case presentation

**Fig. 1:**
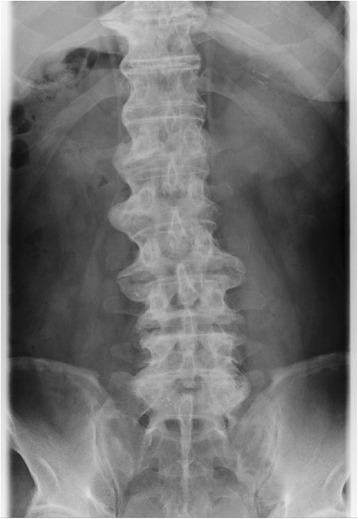
Plain radiographs of the lumbar spine. Antero-posterior view shows non-specific, mild to moderate degenerative changes

A 76-year-old man, with a bladder cancer diagnosis, was scheduled for transurethral resection of a bladder tumor under spinal anesthesia. He weighed 68 kg with a height of 168 cm (BMI = 24.1 kg/m^2^). The patient was previously diagnosed with chronic obstructive pulmonary disease and treated with the inhaled steroid, fluticasone; 250 μg every 12 h, for approximately 3 years prior to admission. A lumbosacral spine x-ray showed age-related mild to moderate cartilage degeneration (Fig. 1).

Spinal anesthesia was administered by a board certified anesthesiologist of the Japanese Society of Anesthesiologist (TK). With the patient in the right lateral position, lumbar puncture was performed at L3-4 inter-space with a 25-gauge Quincke needle *via* a midline approach. Although the click of dural puncture was felt, cerebrospinal fluid (CSF) could not be tapped in two repeated attempts. The spinal needle was checked for deformation or obstruction by tissue and blood clots, but none were observed. A subsequent was made with a 23-gauge needle at L2-3 interspace. After removal of the stylet, backflow of CSF was not detected even after aspiration using a 2.0 ml syringe. After discussion, the anesthesiologist decided to proceed with general anesthesia instead, to which the patient also agreed. Subsequent, anesthesia and surgery proceeded uneventfully. On postoperative day one, the patient complained of moderate intensity headache mainly located in the occipital region, and aggravated by sitting posture. On examination, it was found that except postural headache, there were no associated complaints of vomiting, neck rigidity, or localized signs. It was therefore diagnosed as a case of postdural puncture headache (PDPH). The PDPH was managed conservatively with fluids, bed rest, oral acetaminophen, nonsteroidal anti-inflammatory medications, and oral caffeine. The patient’s headache gradually subsided by the 4^th^ postoperative day. The patient had no neurological complaints or symptoms throughout his hospitalization and was discharged on postoperative day 6.

**Fig. 2:**
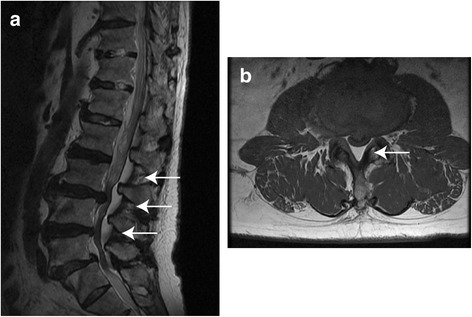
Magnetic resonance imaging T1 weighted view. **a** A sagittal scan shows hypertrophy of epidural fat predominately located posterior from L5 to S1 level. **b** A axial scan of L3-L4 revealed excessive amount of fat within the epidural space compressing the thecal sac. A high signal intensity lesion corresponds to fat (*white arrows*)

Approximately 2 months after discharge, the patient reported progressive lower back pain associated with left leg weakness and numbness. The magnetic resonance imaging (MRI) revealed an epidural mass located at the posterior aspect of the spinal canal extending from the level of L3 to S1 (Fig. 2a). The lesion showed increased intensity on T1-weighted images, compatible with fat. The axial image demonstrated an epidural fat compression of the thecal sac (Fig. 2b). Based on these findings, the patient was diagnosed with SEL. A surgical procedure was carried out on the laminectomy from L3 to L5 and the pathology was confirmed as SEL. At a 12 month of follow-up, the short-term result was promising, he remained asymptomatic.

## Discussion

In this report, we described a case of difficult spinal anesthesia associated with SEL. The patient repeatedly underwent lumbar puncture performed by the trained anesthesiologist; however, adequate CSF back flow could not be obtained. Spinal anesthesia was therefore converted to general anesthesia instead. Postoperatively the patient developed mild but typical PDPH.

Since PDPH is thought to be caused by continuous CSF leakage [7], the failure to obtain CSF in this case may have occurred despite successful dural puncture, rather than technical reasons. The failure of CSF flow before spinal drug administration (often known as a dry tap), is usually caused by a needle blockage, a needle in the wrong space, previous spinal surgery, or low CSF pressures [8, 9]. It can also be secondary to congenital or acquired lumbar canal stenosis, as well as adhesive arachnoiditis [10]. SEL is a pathological condition in which fat tissue is deposited in the spinal canal around the thecal sac [1–3]. This excessive deposition in the epidural space may obliterate the spinal subarachnoid space, which could lead to low CSF pressure and in this instance would explain the dry tap.

The most common cause of SEL has been reported to be oral administration with exogenous steroids [1–3]. The incidence of SEL is particularly pronounced after regimens involving higher dosages and in those undergoing long-term treatment. In this case, the patient has been using relatively high-dose inhaled steroid for approximately 3 years prior to admission, which may have resulted in the development of SEL. There have been similar case reports of patients developing SEL in association with long-term use of inhaled steroids [11]. On the other hand, SEL has also been described in patients with morbid obesity, as well as in those with no identifiable risk factors [1–4]. Therefore, the possibility of non–steroid cause of SEL, including obesity-related and idiopathic case, cannot be excluded in our patient.

MRI is thought to be the best modality for diagnosis of SEL and high-signal intensity on T_1_-weighted images is characteristic of adipose tissue [1–3]. Epidural fat with a thickness of greater than 7 mm has been reported to be the diagnostic for SEL [12]. Nevertheless, SEL cannot be diagnosed on plain x-ray films. Therefore, as in our case, it is difficult to identify on routine preoperative examination if the symptoms are not typical.

## Conclusion

Our case demonstrates that SEL may be associated with difficult lumbar puncture due to a dry tap.

## Abbreviations

CSF, cerebrospinal fluid; MRI, magnetic resonance imaging; PDPH, postdural puncture headache; SEL, spinal epidural lipomatosis.
